# HeliantHOME, a public and centralized database of phenotypic sunflower data

**DOI:** 10.1038/s41597-022-01842-0

**Published:** 2022-11-30

**Authors:** Natalia Bercovich, Nikita Genze, Marco Todesco, Gregory L. Owens, Jean-Sébastien Légaré, Kaichi Huang, Loren H. Rieseberg, Dominik G. Grimm

**Affiliations:** 1 Department of Botany, University of British Columbia, Vancouver, British Columbia Canada; 2 Biodiversity Research Centre, University of British Columbia, Vancouver, Canada; 3 Technical University of Munich, Campus Straubing for Biotechnology and Sustainability, Bioinformatics, Straubing, Germany; 4 Weihenstephan-Triesdorf University of Applied Sciences, Straubing, Germany; 5 Department of Biology, University of Victoria, Victoria, BC Canada; 6 Department of Computer Science, University of British Columbia, Vancouver, British Columbia Canada; 7 Data Science Institute, University of British Columbia, Vancouver, British Columbia Canada; 8 Technical University of Munich, Department of Informatics, Garching, Germany

**Keywords:** Plant sciences, Genome-wide association studies, Databases

## Abstract

Genomic studies often attempt to link natural genetic variation with important phenotypic variation. To succeed, robust and reliable phenotypic data, as well as curated genomic assemblies, are required. Wild sunflowers, originally from North America, are adapted to diverse and often extreme environments and have historically been a widely used model plant system for the study of population genomics, adaptation, and speciation. Moreover, cultivated sunflower, domesticated from a wild relative (*Helianthus annuus*) is a global oil crop, ranking fourth in production of vegetable oils worldwide. Public availability of data resources both for the plant research community and for the associated agricultural sector, are extremely valuable. We have created HeliantHOME (http://www.helianthome.org), a curated, public, and interactive database of phenotypes including developmental, structural and environmental ones, obtained from a large collection of both wild and cultivated sunflower individuals. Additionally, the database is enriched with external genomic data and results of genome-wide association studies. Finally, being a community open-source platform, HeliantHOME is expected to expand as new knowledge and resources become available.

## Abstract (summary)

**Table:** 

Measurement(s)	plant trait
Technology Type(s)	ImageJ
Factor Type(s)	Country • Sitename • Latitude • Longitude • Elevation
Sample Characteristic - Organism	Helianthus annuus var. macrocarpus • Helianthus annuus • Helianthus argophyllus • Helianthus niveus subsp. canescens (taxid: 74145) • Helianthus petiolaris subsp. fallax (taxi: 74150) • Helianthus petiolaris subsp. petiolaris (taxid: 74151)
Sample Characteristic - Location	United States of America • Canada

## Background & Summary

The sunflower genus comprises more than 50 wild species and 19 subspecies^1,2^ all of which are native to North America. Wild sunflowers colonize all kinds of extreme environments, from sand dunes to coastal salt marshes, displaying remarkable inter- and intra-specific plasticity and ability for adaptation. Moreover, the common sunflower, *Helianthus annuus* var. *macrocarpus*, is one of the seven major oilseed crops produced around the world^3^. The study of natural variation in wild relatives of crops like sunflower offers opportunities to increase our understanding of their evolutionary history and adaptation to different environments, as well as to enhance breeding programs. In the past decade, large efforts from numerous research groups around the globe have completed the sequencing and assembly of the common sunflower genome as well as other genomic and germplasm resources^4–8^. The current availability of several high-quality reference genomes for sunflower has made it possible to start asking new types of questions about sunflower molecular ecology and its evolutionary history^6–8^. It also opens the possibility to broadly investigate genetic associations between sequence and gene function in a more accurate way (e.g. Duriez *et al*.; Todesco *et. al*)^9,10^.

**Fig. 1:**
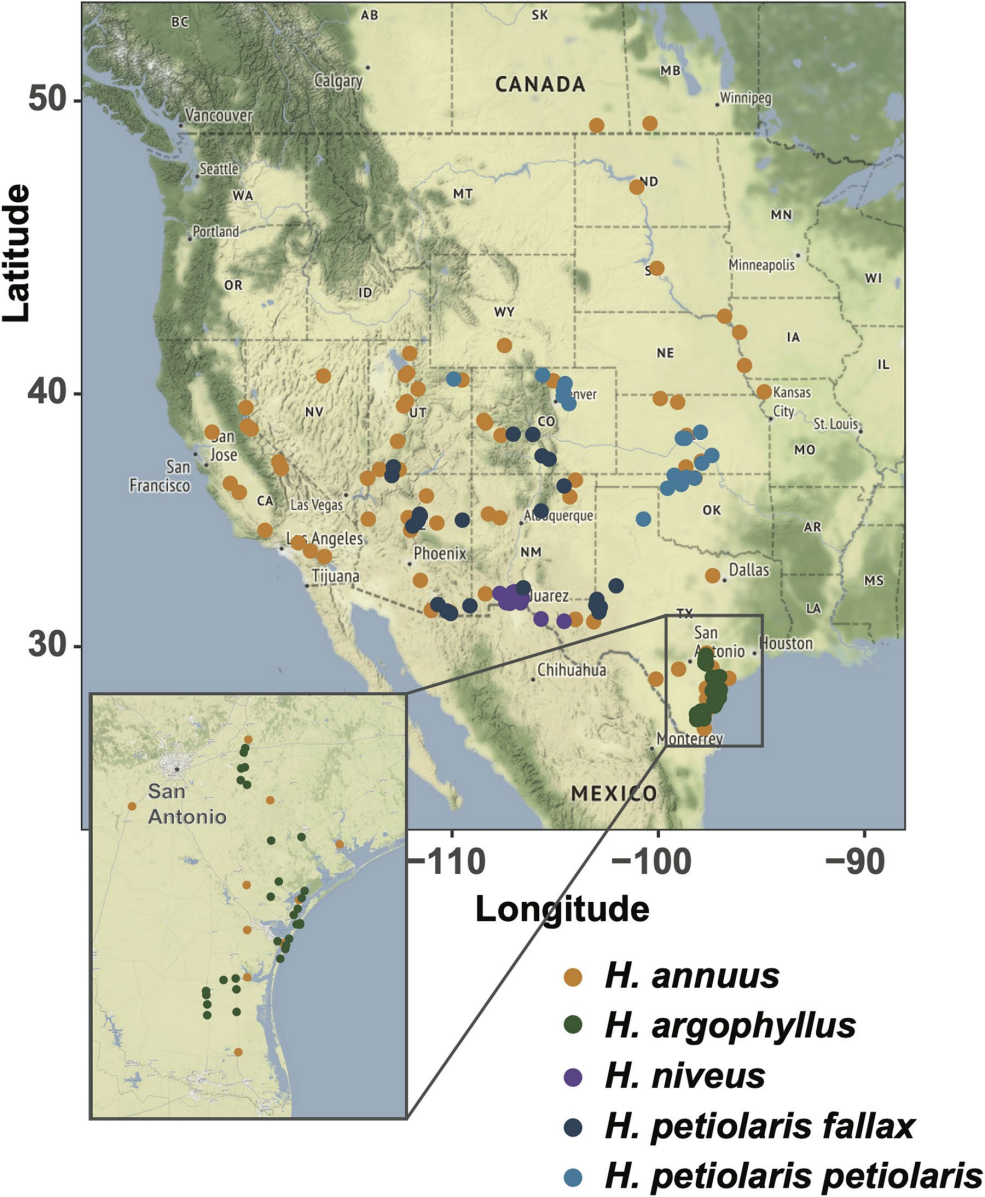
Distribution of wild sunflower populations. Section of a map from North America showing the distribution of the studied populations of wild sunflowers included in HeliantHOME, corresponding to four different species: Helianthus annuus depicted in orange, Helianthus argophyllus shown in green, Helianthus niveus shown in purple and two subspecies of Helianthus petiolaris, highlighted in different shades of blue.

We have recently carried out a comprehensive study of the genetic and phenotypic diversity as well as of their association with environmental variables for four annual wild sunflower species: *H. annuus*, *H. petiolaris, H. niveus* and *H. argophyllus*^6^. These species were selected based on their ability to grow in a broad variety of – often extreme – environments and their potential use for sunflower breeding. Populations for these four species are found all across the US (see Fig. 1), coexisting at times, and showing adaptation to diverse environments often rising to the ecotypic level (subpopulations within the same species).

In a recent work, we carried out a common garden experiment at a single location in Vancouver, Canada and collected phenotypic data for 1,510 individuals belonging to 151 populations for the wild sunflower species mentioned above. We additionally re-sequenced the genomes of most of those individuals, resulting in sets of >4 M high-quality single nucleotide polymorphisms (SNPs) for each of the four species. Given the broad relevance of these datasets, for both the sunflower research and breeding communities, we decided to generate HeliantHOME, an interactive database that contains all these data in a way that can be easily reused, revisited and even enriched by the research community. One of the features we kept into consideration was universality and reproducibility of data. Therefore, whenever possible, we aimed to follow all the ‘minimum information about plant phenotyping experiments’ standards checklist^11,12^ (MIAPPE: https://www.miappe.org/), to ensure we could offer the most adequate data, including unique identifiers, detailed descriptions of experimental procedures, measurements, units, and others. Also, we provide data in the most commonly used data formats, such as PLINK and CSV files for GWAS analysis and more importantly, the REST API which conveniently allows to access additional meta-data.

Both raw and extracted phenotypic data have been incorporated in the database; this includes a large collection of high-resolution images of different plant organs, architectural and developmental trait measurements as well as complementary environmental variables (climate and soil data) for the population of origin of the individuals included in the datasets. The corresponding re-sequencing data for most of the individuals is also linked and available for public download.

In addition, we have included in HeliantHOME phenotypic data for the UGA-SAM1 (SAM) population, a collection of 288 cultivated sunflower lines that have been selected because they capture nearly 90% of the allelic diversity present within cultivated sunflower^13^. Most individuals represent modern oil or confectionary cultivars, however, a handful of open-pollinated varieties, landraces and prebred lines are also included. This collection is publicly available and it can be retrieved from both the Agricultural Research Service from the US Department of Agriculture (USDA-ARS)^14^, and the French National Institute for Agricultural Research & Environment (INRAE)^15^. These individuals are propagated and maintained as isogenic lines for research purposes and became a standard tool for the study of phenotypic variation and adaptation in sunflower^16–20^. A list of the SAM population lines is available in Supplementary Table 1.

The two datasets provided in HeliantHOME (wild sunflowers and SAM population) offer complementary information on sunflower diversity and its potential use for crop improvement. Many genes that confer adaptation to extreme environments or disease in cultivated sunflower have been introgressed from wild sunflower populations^13,21–23^ and often, close wild relatives are the source of novel and advantageous alleles sought by the agricultural sector^2^. Thus, the wild sunflowers dataset contained in HeliantHOME represents an unparalleled tool. However, the wild sunflower individuals that we have genotyped could not be maintained (wild sunflowers are obligated outcrossers) and consequently, no further phenotypic information can be added to the dataset.

The SAM population instead, is composed of inbred lines, which can be grown repeatedly in different conditions. The SAM phenotypic dataset is therefore in continuous expansion and constitutes a powerful complementary tool for studies looking at the genetic basis of phenotypic diversity and domestication in sunflowers.

HeliantHOME includes, among others, a rich dataset of high-quality images for individual plants and plant organs, arguably making it one of the finest and most extensive collections of population scale phenotypic data of its kind existing so far. In addition to its obvious utility for the sunflower research community, this collection could as well be suitable as a labeled high-quality dataset, for the development of novel machine learning methods for automatic phenotype extraction or computer vision in general.

Previous experience has shown the usefulness to the scientific community of a curated database like the one we are presenting. AraPheno^24^, a similar public database for phenotypic data in *Arabidopsis thaliana* as well as AraGWAS^25,26^, a manually curated and standardized GWAS (Genome Wide Association Studies) catalog, have been recently developed and both have been broadly used and expanded since. Once data is centralized and publicly shared, new discoveries can emerge and new analyses become possible.

In summary, HeliantHOME will be a fundamental tool for researchers coming from different fields and not necessarily working with sunflower. It offers a broad dataset for both basic and applied plant sciences, from an evolutionary, ecological and comparative genomics perspective to a computational and even machine learning standpoint.

## Methods

### Wild sunflower data

**Fig. 2:**
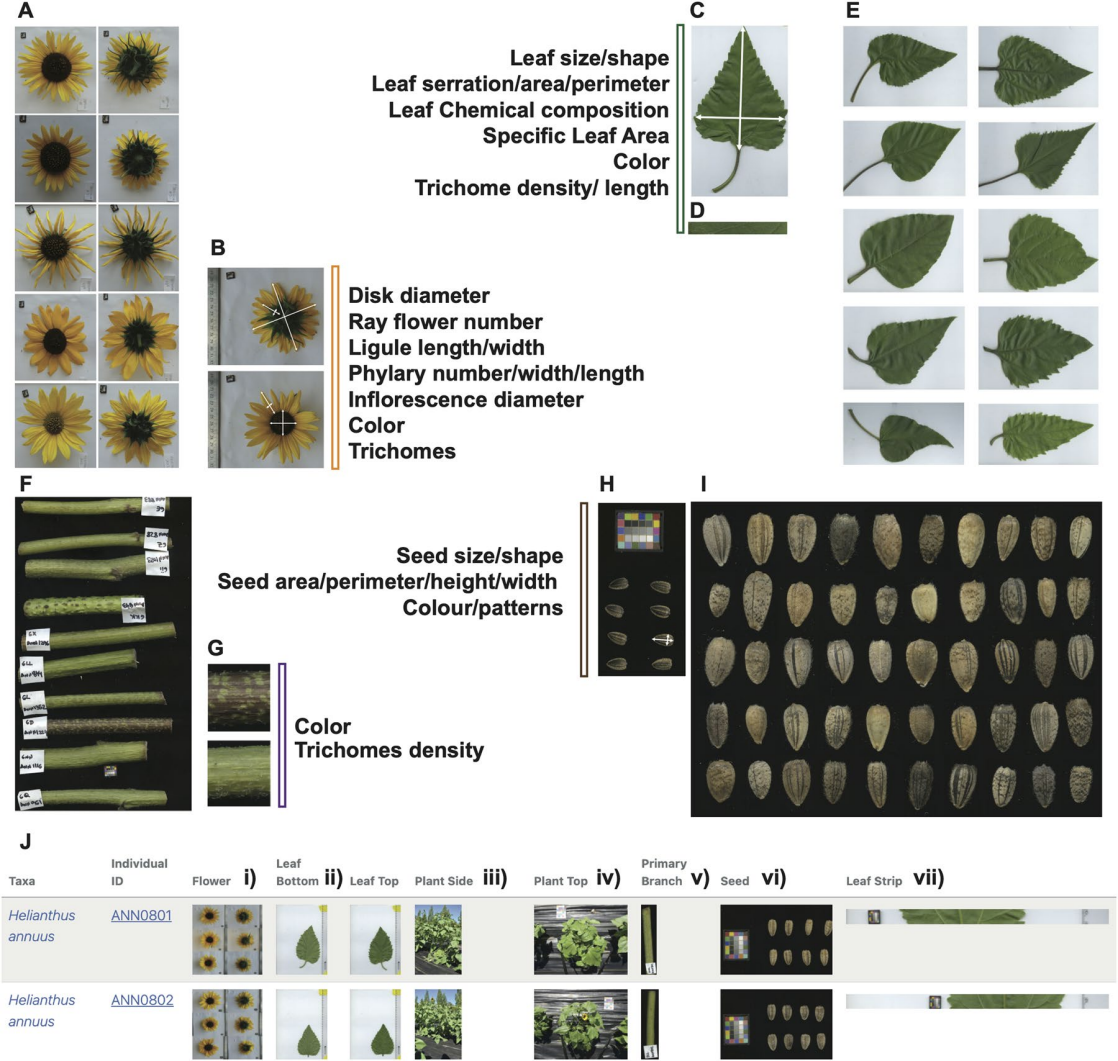
Helianthus annuus morphological diversity and the associated high-resolution images available in HeliantHOME. (**A**) Assorted selection of H. annuus inflorescences images taken from top (left panel) and bottom (right panel) showing morphologic diversity; (**B**) Example of the measurements performed for each single inflorescence using Fiji; (**C**) Measurements recorded using Fiji for leaf traits; (**D**) Example of a high-resolution scan of a leaf section used to detect trichome density (**E**) Assorted selection of leaf images as an example of the morphological diversity observed in the wild; (**F**) Assorted selection of primary branches sections as an example of the amount of variation observed; (**G**) Close up for two different branches and description of the measured traits; (**H**) Scanned image obtained for each single individual of eight randomly selected seeds. (**I**) Assorted selection of seeds showing variability observed in the wild for this trait. (**J**) Screenshot from HeliantHOME images section, example showing for each individual (in this case IDs: ANN0801 and ANN0802 (i) the first three inflorescences both from top/bottom (camera captured); (ii) a single young, fully expanded leaf at anthesis both adaxial and abaxial sides (scanned); (iii) lateral image of the whole young plant before flowering (camera captured); (iv) image captured from above of the whole young plant (camera captured); (v) section of primary branch (scanned); (vi) random selection of 8 seeds coming from a cross within members of the same population (scanned at 2400 dpi high resolution) and (vii) 2400 dpi high resolution scan of a section of the abaxial side of the same leaf recorded in ii).

In the summer of 2016 ten mother plants were randomly selected from 151 populations of wild sunflowers collected in previous years (2011 and 2015) from across the US and southern Canada, for four sunflower species: *H. annuus*, *H. petiolaris*, *H. niveus* and *H. argophyllus*. Seeds from each of these plants were germinated, and eventually transplanted into three separate fields at the Totem Plant Science Field Station of the University of British Columbia, Vancouver campus, Vancouver, Canada. Within each field, pairs of plants from the same population were sown using a completely randomized design. Phenotypic measurements were assessed daily throughout plant development, and leaves, stem sections, inflorescences and seeds were collected and digitally recorded for further analyses. We measured a total of up to 87 different traits per individual which can be divided in four main categories: 1) Plant Development & Architecture: including traits related to plant growth, days to flowering, number of primary branches, final height, etc. These traits were measured manually in the field, using precision tools. 2) Inflorescence Traits; including traits, such as flowerhead diameter, number of ligules (petals of the outermost whorl of flowers in the inflorescence circumference), etc. In this case, high resolution images were recorded and eventually analyzed using the Fiji software^27^ (see Fig. 2A,B).

3) Leaf and branch traits: including traits like leaf area, C/N content, trichomes density, etc. Most leaf traits required the use of scanned high-resolution images and eventual digital analysis (see Fig. 2C,G), using Tomato analyzer software or Fiji^27,28^. For C/N content, we collected and ground tissue, and submitted samples to EA-IRMS (Elemental analyzer isotope ratio mass spectrometry) analysis at the Stable Isotope Facility, Faculty of Forestry, UBC, Vancouver Canada. 4) Seed traits: including traits like seed height, perimeter, etc. The seeds were obtained from restricted crosses (only crosses within individuals from the same population) carried out using pollination bags. Seeds were eventually harvested when dry and scanned at high resolution. Images were captured for further digital analysis^28^ (see Fig. 2H,I).

Detailed information about methods used for the collection of phenotypic developmental data or for the morphometrics obtained from the associated image analyses are provided in Supplementary Table 2. The table includes phenotype name, methodological tools and a brief description of the procedures. More information can also be found in the original publication^6^.

In summary, we provide a) developmental data obtained from this experiment by direct observation of live plants; b) data associated with architecture and development of the plants (by indirect measurement performed on recorded images) as well as c) the high-resolution color images collected for all the studied individuals and their harvested organs.

### Cultivated sunflower data

The UGA-SAM1 (SAM) population phenotypic data available in the sunflower community is a collection in continuous expansion that has been growing for the past 10 years, with data obtained both in greenhouse and field settings. We have for now added data for one study carried out in a greenhouse setting to illustrate the potential benefits of including these data in HeliantHOME and are working on the inclusion of a larger dataset^16,17^. The evaluated phenotypes for the cultivated individuals currently available are described in Table 1.

**Table 1:** SAM population phenotypes.

Phenotype ID	Measurement method
**Leaf Traits**	
LA (leaf area)	Recently expanded leaves were scanned against a white background with a ruler in the image; images were transformed into binary (black/white) in Image J; area was measured with reference to the scale object using the Wand (tracing) tool
LMA (leaf mass per area)	Ratio of dry leaf mass to fresh leaf area (measured as above using a scanned image). After scanning (with a standard resolution of 300DPI), leaves were dried for at least 3 days at 60°C and then weighed
CHL (chlorophyll content)	Measured using SPAD-502 meter, Konica Minolta. The average of four measurements per pair of recently expanded leaves per plant was taken (absorbance ratio)
**LNmassC**	
(Leaf Nitrogen Composition)	A recently fully-expanded leaf on each plant was collected, scanned, and then dried at 60 °C to constant mass. The dry leaves were ground with ball mill to fine powder, then dried again at 60 °C before weighing out 2–5 mg for Nitrogen analysis with an isotope ratio mass spectrometer (PDZ Europa Integra, Cheshire, England), which estimates total elemental concentrations, in UBC Forestry Stable Isotope Facility. Nmass (%): mass-based nitrogen concentration
LN (leaf number)	Number of true leaves >2 mm long
**Plant Architecture**	
PH (Plant height)	The length along stem from the soil surface to the apex
SD (Stem diameter)	Measured at the first leaf node nearest base of stem with calipers; took the maximum from 3 measurements as many stems were not perfectly cylindrical
HD (Hypocotyl diameter)	Measured at base of stem above soil with calipers; took the maximum from 3 measurements
SB (Shoot biomass)	Harvested all above-ground biomass and placed into labelled paper bag(s). The plants were dried at ~60°C for at least 3 days, or until biomass reached a constant weight, then weighed (in g)
**Roots traits**	
ARN (Number of adventitious roots)	All new roots from hypocotyl longer than 1 mm were counted

Detailed methodological description of the traits measured for the SAM population in a GWAS flooding experiment carried out by Gao *et al*. in 2019^17^.

### HeliantHOME

**Fig. 3:**
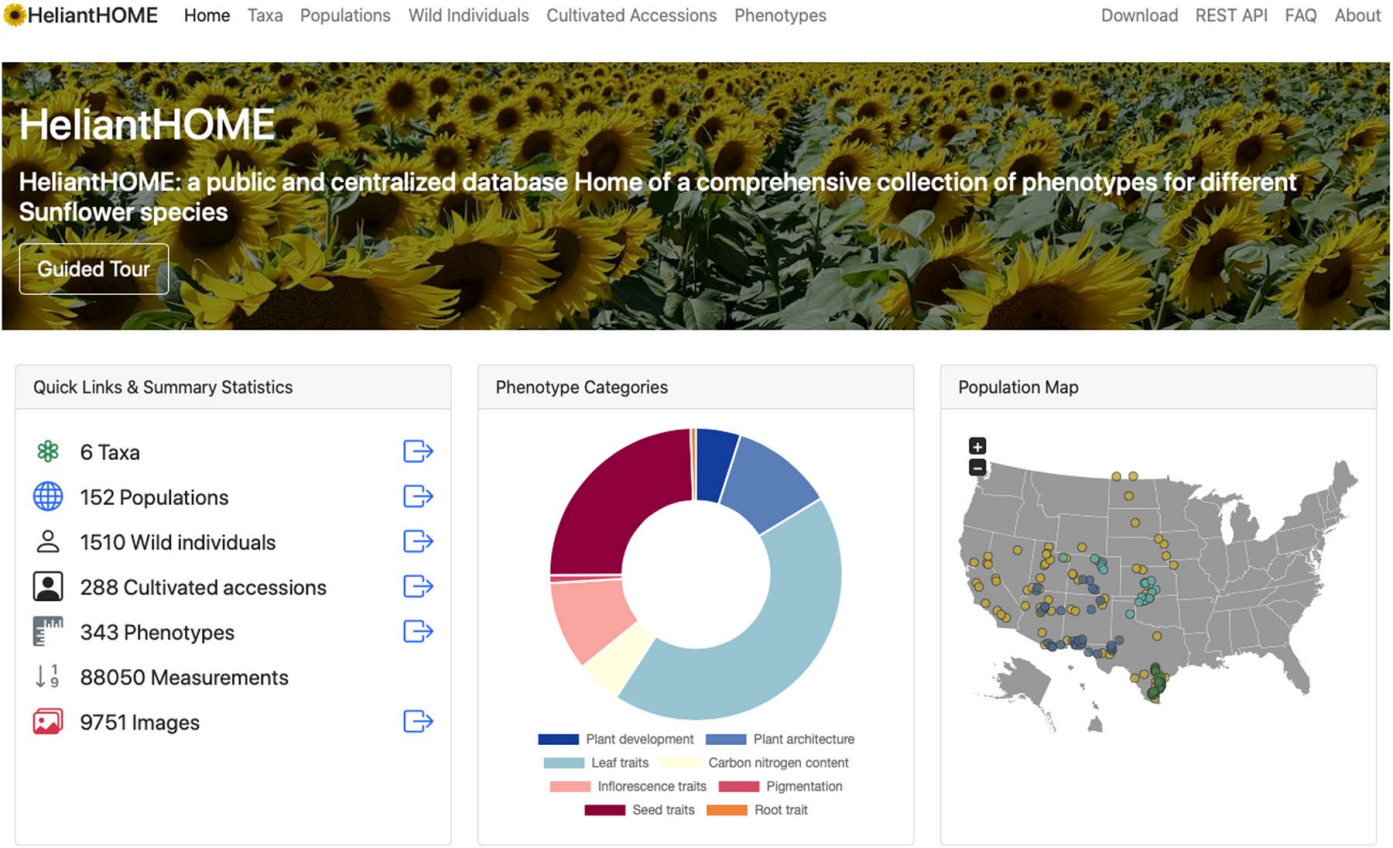
HeliantHOME Landing page features. Screenshot of the landing page of HeliantHOME (http://www.helianthome.org). At the top, a navigation menu is provided to quickly navigate to different views. In addition, links to direct database downloads, FAQs and the REST API are available on the right side of the navigation menu. At the bottom different summary statistics are shown and illustrated with dynamic JavaScript plots. On the bottom right corner, an interactive map shows the distribution of the characterized populations.

To facilitate exploration, search, filtering and download of phenotypic as well as meta-data we developed the public web-application HeliantHOME (http://www.helianthome.org). The primary purpose of HeliantHOME is to simplify data access and to provide detailed information about the different sunflower species, populations, phenotypes and associated images. The database can be easily queried via a public web-interface as well as programmatically crawled via the implemented public Representational State Transfer (REST) interface. Detailed FAQs, tutorials and guided tours are implemented to guide novice users and to help them navigate through the different views. Figure 3 shows a screenshot of the landing page of HeliantHOME.

**Fig. 4:**
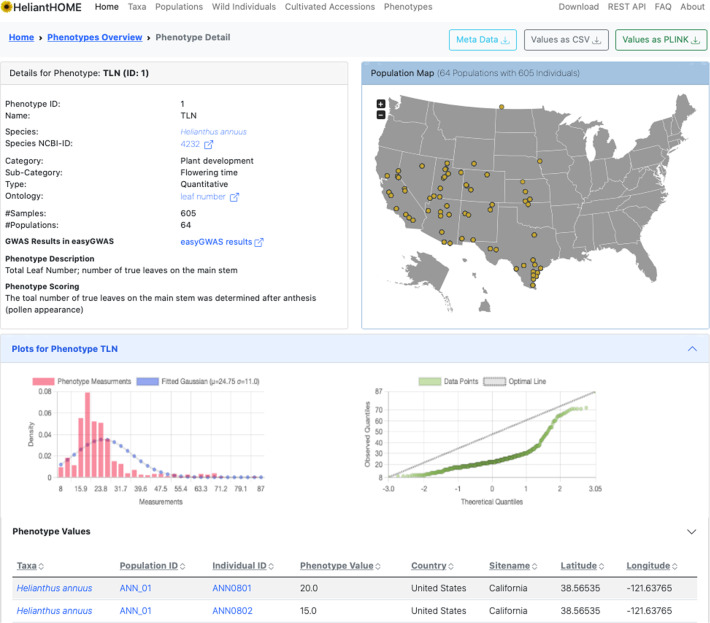
Phenotypes overview. Screenshot of the detailed view for a certain phenotype (http://www.helianthome.org/phenotype/1/). General information about the phenotype is shown in the left top corner of the screenshot. A detailed population map shows the distribution of samples for which we measured this phenotype within the US. Additional interactive plots are provided to investigate the phenotypic distribution. At the top right, different download options are provided to download the phenotypic values in different formats as well as all the available meta-information.

Various views are provided to summarize information about the different species, populations and phenotypes. All integrated data is associated with additional meta-information. For example, the different sunflower populations are linked with location information (latitude and longitude), as well as detailed information about climate and soil variables. The phenotype view summarizes detailed information about the phenotype scoring, as well as additional meta-information and interactive visualizations to analyze the distribution of phenotypic values (see screenshot in Fig. 4). In addition, phenotypes are linked to genome-wide association study (GWAS) results, available at easyGWAS^29^. The database also hosts high-resolution imaging data for all wild sunflower individuals (http://www.helianthome.org/images/). All displayed information and data can be easily downloaded by the user in various data formats, such as CSV, PLINK, JSON or as imaging files. An integrated REST API allows fast, scalable and customizable programmatic access to the data. Further, additional external resources to genetic data are linked in the Download Center of HeliantHOME (http://www.helianthome.org/download/), to allow the simple download of associated material.

### Implementation

HeliantHOME is a public and manually curated database implemented using the open-source Python Django (v.3.2.4) web-application framework (https://www.djangoproject.com). The database backend is based on SQLite (https://www.sqlite.org/index.html). Several third-party Python libraries have been included to allow efficient filtering of the database as well as to provide common data handling and statistical analysis methods like numpy^30^, scipy^31^ and pandas^32^.

In addition, the Django REST framework (https://www.django-rest-framework.org) has been used to implement various REST endpoints to simplify the programmatic access and download of all the data stored in HeliantHOME. A detailed documentation of all REST endpoints can be found here: http://www.helianthome.org/rest/api.

The web-application frontend is based on HTML5 and Bootstrap (https://getbootstrap.com), a modern and responsive CSS framework. The interactive parts are implemented using jQuery (https://jquery.com). Dynamic visualizations and plots are integrated and are based on the JavaScript libraries Chart.js (https://www.chartjs.org) and jVectorMap (https://jvectormap.com).

To guide novice users through the web interface and to introduce the different views a fully guided and interactive tour is provided using intro.js (https://github.com/usablica/intro.js).

The code for HeliantHOME is open-source and hosted on GitHub. Further, the repository includes a detailed list of all used packages and allows users to report issues or to submit feedback: https://github.com/grimmlab/HeliantHome.

## Data Records

In addition to HeliantHome, all phenotypic data and images are also deposited at the digital library of the Technical University of Munich (10.14459/2022mp1649709)^33^. The data consists of a large collection of high-resolution images and extracted phenotypic measurements for 1,510 wild sunflower individuals and 288 cultivated sunflowers. All the data provided here have been previously published and/or is currently public^6^. We have a set of recorded data for 343 different phenotypes and 88,050 measurements; see Table 2 for additional featured data.

**Table 2:** HeliantHOME statistics.

Data Content	Data Statistics
Species	6
Populations	152
Wild Individuals	1,510
Cultivated Accessions	288
Phenotypes	343
Phenotypic Measurements	88,050
Phenotype Categories	8
High Resolution Plant Images	9,751

Data content and statistics at the database as of 15^st^ of March 2022.

HeliantHOME is a modern responsive web interface that allows easy access, filtering and download of phenotypic and complementary genotypic sunflower data for a large collection of both wild and cultivated sunflowers.

Among the different features, HeliantHOME holds 9,751 high resolution images corresponding to the wild sunflower individuals and their different organs, as illustrated in the screenshot shown in Fig. 2J.

The data stored at the digital library of Munich allows the download of the full dataset at once, whereas HeliantHome allows the specific download of individual data entries via the included REST API, as well as the download of custom imaging data for certain individuals.

## Technical Validation

### Wild sunflower data

The common garden experiment involved fully randomized (in pairs) planting of all the individual plants to avoid location effects. In addition, assorted wild sunflower plants were planted all around the edges of the field to minimize border effects. Standard procedures were used to determine developmental traits. Restricted pollination was used to produce seeds while avoiding cross-pollination from different populations. Unlike cultivated sunflower, wild sunflower is self-incompatible.

## Usage Notes

Phenotypic and imaging data can be downloaded directly from the digital library of the Technical University of Munich (10.14459/2022mp1649709)^33^, conveniently from HeliantHOME using the web-interface or via custom Python scripts using the publicly available REST interface. Various statistical methods can be used to analyse the phenotypic data. A primary example are genome-wide association studies (GWAS). For this purpose, univariate association tests (associations between single point-mutations with a certain phenotype) that account for population structure, such as FaSTLMM (Factored Spectrally Transformed Linear Mixed Models)^34^ or permutation based GWAS^35^ can be used. The data might also be used for the comparison or development of new phenotype prediction methods. The imaging data can be analyzed using custom Python scripts and might also serve the computer science community to develop novel machine learning and computer vision methods for automatic phenotyping^36,37,38^.

## Supplementary information

Supplementary Table 1

Supplementary Table 2

## Data Availability

All code for the web server backend and frontend are publicly available for download on GitHub: https://github.com/grimmlab/HeliantHome.The web-application can be accessed via: http://www.helianthome.org.
